# Pancreatic metastasis of Merkel cell carcinoma and concomitant insulinoma: Case report and literature review

**DOI:** 10.1186/1477-7819-3-58

**Published:** 2005-09-01

**Authors:** Jeannine Bachmann, Jorg Kleeff, Frank Bergmann, Shailesh V Shrikhande, Wolfgang Hartschuh, Markus W Büchler, Helmut Friess

**Affiliations:** 1 Department of General Surgery, University of Heidelberg, Heidelberg, Germany; 2 Department of Pathology, University of Heidelberg, Heidelberg, Germany; 3 Department of Dermatology, University of Heidelberg, Heidelberg, Germany

## Abstract

**Background:**

Merkel cell carcinomas are rare neoplasm of neuroendocrine origin, usually observed in elderly people in areas with abundant sunlight, and predominantly located on the head and neck, extremities, and trunk. In many patients, a local recurrence after resection of the primary tumour and even distant metastases can be found.

**Case presentation:**

We report an unusual occurrence of pancreatic metastases from a previously diagnosed Merkel cell carcinoma with the discovery of a concomitant insulinoma. An 82-year old lady suffered from recurrent attacks of hypoglycemia and presented with an abdominal mass, 2 years prior she had an excision done on her eyebrow that was reported as Merkel cell carcinoma. An extended distal pancreatectomy and splenectomy along with resection of the left flexure of the colon for her abdominal mass was carried out. Final histopathology of the mass was a poorly differentiated endocrine carcinoma in the pancreatic tail, in the peripancreatic tissue and in the surrounding soft tissue consistent with metastatic Merkel cell carcinoma in addition to an insulinoma of the pancreatic body.

**Conclusion:**

This is the first documented case of a metastatic Merkel cell carcinoma and a concomitant insulinoma, suggesting either a mere coincidence or an unknown neuroendocrine tumor syndrome.

## Background

Merkel cell carcinoma is a rare cutaneous neoplasm of neuroendocrine origin, usually observed in elderly people in areas with abundant sunlight. Affected skin areas are predominantly the head and neck, but the extremities and trunk are other predisposed areas [1-4]. In a review of 1024 patients, the primary tumour was found on the head and neck in 40% of the patients, in 33% on the extremities and in 23% on the trunk [5]. In 98% of reported cases this cancer could be found in Caucasians, suggesting possible protection by darker skin pigmentation [5]. While the exact incidence remains unknown, Hodgson found an increase in the incidence of Merkel cell carcinoma from 0.15 cases per 100,000 in 1986 to around 0.44 cases per 100,000 in 2001; the highest incidence of 4.28 per 100,000 was found in patients over 85 years [6]. The literature review of 1024 patients showed a mean age of 69 years at diagnosis [5]. We report an unusual occurrence of pancreatic metastases from a previously diagnosed Merkel cell carcinoma with the discovery of a concomitant insulinoma.

## Case presentation

**Figure 1:**
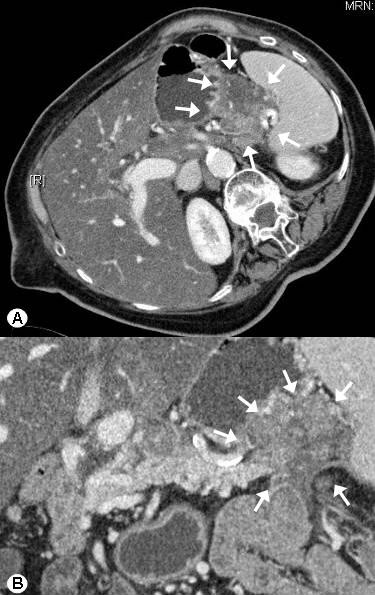
Computed tomography scan of the abdomen revealed a large lesion of 5 to 6 cm in relation to the pancreatic body and tail (venous phase A, B, arrows).

**Figure 2:**
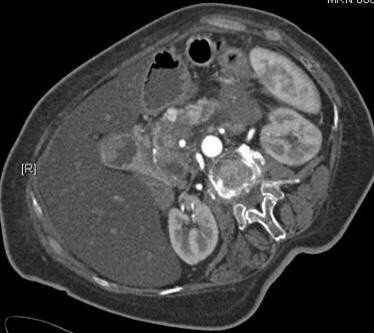
Arterial phase of the computed tomography scan of the abdomen shows a hypervascularized area (arrows) in the pancreatic body.

A 82-year old lady presented to the Department of General Surgery at the University of Heidelberg, Germany with recurrent attacks of hypoglycemia and a large abdominal mass. While diagnostic tests repeatedly documented glucose levels below 40 mg/dl (normal levels 80 – 120 mg/dl), a computed tomography (CT) (Figure 1A, B) scan of the abdomen revealed a large lesion of around 5 to 6 cm in relation to the pancreatic body and tail. There were also large masses of about 3–5 cm in the retroperitoneum and in the area of the celiac trunk and around the mesenteric artery. Furthermore, in the pancreatic body there was a hypervascularized area (Figure 2), that was suspicious for an insulinoma. Clinically this lady, who was not thriving, reported a weight loss of 12 kilograms over the previous 4 months. A somatostatin receptor scintigraphy showed an enhanced uptake in the region of the pancreatic body/tail as well as in the right axilla (a palpable mass was also noted there) and excluded the possibility of other involved areas.

**Figure 3:**
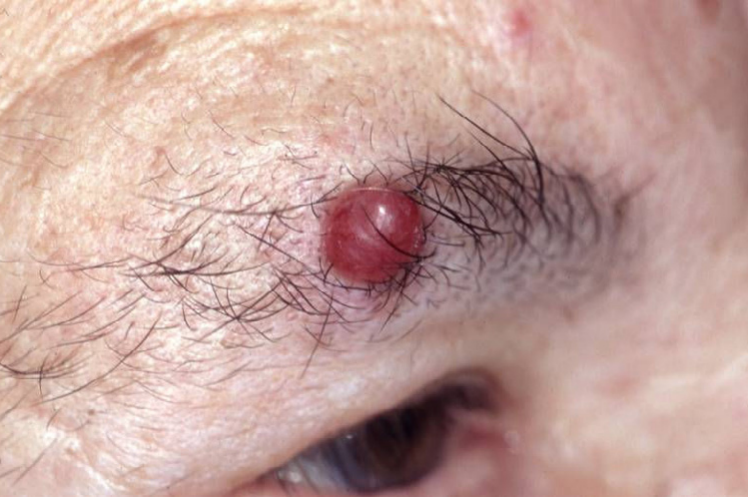
Merkel cell carcinoma on the right eyebrow.

**Figure 4:**
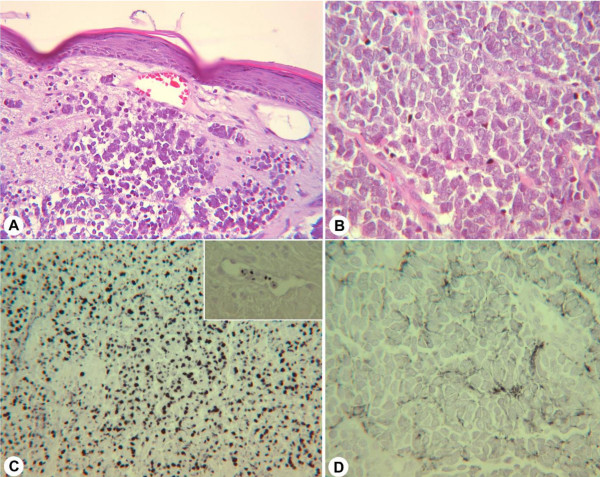
Merkel cell carcinoma – primary tumor. A, B: Hematoxylin and eosin staining. A. Lower magnification showing intravasal tumor cells. B: Monomorphous tumor cells, pale staining nuclei, many mitotic figures. C: Strong cytokeratin 20 staining. Insert: Intravasal cytokeratin 20-positive tumor cells. D: Weak chromogranin A staining

She gave a past history of an operation done on the right eyebrow 2 years prior for a 0.8 × 0.8 cm lesion (Figure 3) that was reported as a Merkel cell carcinoma. Histopathology showed rather uniform tumor cells in a trabecular growth pattern (Figure 4a) with monomorphous pale-stained nuclei and many mitoses (Figure 4b). There was invasion of dermal lymphatics and blood vessels (Figure 4a, c, insert). Immunohistochemistry revealed strong positivity for cytokeratin 20 (Figure 4c) and neurofilament (not shown) in the characteristic dot-like pattern and a weak expression of chromogranin A (Figure 4d). After excision, radiation therapy was also administered only at the site of the primary lesion, the draining lymphatic vessels and the first lymph node station. A year later, a large abdominal mass was noted of uncertain origin and an ultrasound guided biopsy showed an unspecified small cell cancer. In view of the large mass with additional suspicious areas being noted in the spleen, left adrenal gland and axilla, she had been subjected to palliative radiotherapy of 30 Gray over 2 months. However no definitive diagnosis of metastasis in these areas was established. With a working clinical diagnosis of symptomatic insulinoma not responding to medical measures, a decision for surgical resection of this large lesion was inevitable, the age of the patient and the previous history of palliative radiation just 6 months prior notwithstanding.

### Operative details

Surgical exploration revealed a large mass of about 5 cm in the tail of the pancreas, in close proximity to the spleen and the splenic flexure of the transverse colon. However there was no evidence of any metastatic disease to the liver, peritoneum and the adnexae. After a careful and meticulous mobilization, a distal pancreatectomy, splenectomy, and adrenalectomy along with resection of the splenic flexure of the colon were performed.

**Figure 5:**
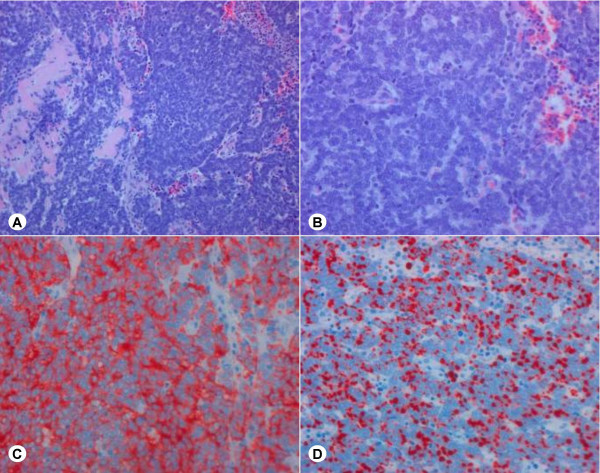
Merkel cell carcinoma metastasis in the pancreas. A, B: Hematoxylin and Eosin staining. C: Synaptophysin staining. D: Cytokeratin 20 staining.

Pathological examination revealed a tumor with manifestations in the pancreatic tail, the adrenal gland, the peripancreatic tissue, and the surrounding soft tissue. Grossly, the mass displayed a whitish and glassy cut surface, containing extended areas of haemorrhage and necrosis. Histologically, the tumor displayed endocrine architecture with mostly solid formations of rather monomorphic cells. The tumor was mitotically highly active (mitotic count >10 per high power field) and contained abundant areas of necrosis. Immunohistochemically, the tumor cells were strongly positive for the endocrine marker synaptophysin and for cytokeratin 20 while there was no expression of insulin. The proliferative activity (MIB-1) reached approximately 80% (Figure 5).

**Figure 6:**
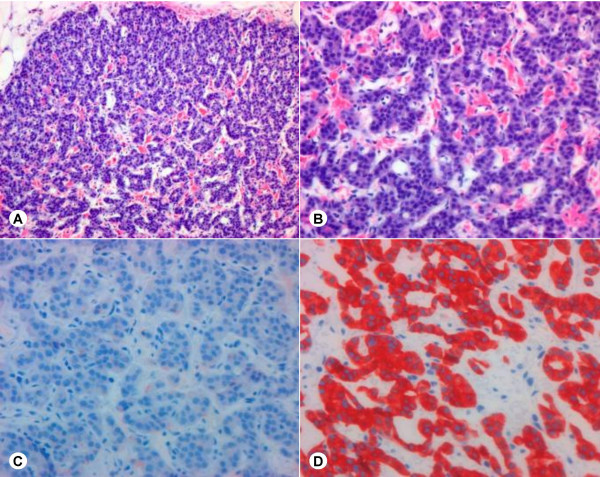
Insulinoma in the pancreatic body. A, B: Hematoxylin and Eosin staining. C: Insulin staining (note the focal positivity). D: Synaptophysin staining.

Furthermore, gross examination of the resected specimen revealed a well demarcated, brownish tumor of the pancreatic body, measuring 1.2 cm in diameter. This tumor microscopically displayed endocrine architecture with trabecular arrangements of uniform tumor cells, showing no mitotic activity. Immunohistochemistry revealed strong positivity for synaptophysin as well as focal positivity for insulin. The proliferative activity (MIB-1) was approximately 1% (Figure 6). The diagnosis of a poorly differentiated endocrine carcinoma (Merkel cell carcinoma) along with that of benign pancreatic insulinoma was thus made.

The patient had a smooth postoperative recovery, the bouts of hypoglycaemia completely disappeared, and she was discharged home within 3 weeks of surgery. She is presently asymptomatic and remains on regular follow up.

## Discussion

Though a very rare tumor, Merkel cell carcinoma is inherently aggressive. The cells of origin are thought to be Merkel cells, which are neuroendocrine cells present in the basal layer of the epidermis. However the exact origin is controversial since often on immunohistological examination both epidermal and neuroendocrine features can be found. This tumor has a high tendency for local recurrence [2,7-9]. While in up to 50% of patients local recurrence after excision is observed, most are diagnosed within two years of the primary resection [1]. 50 – 75% of the patients develop lymph node metastases [5,9-12]. Distant metastases are described in many cases [1,5,7,8,13]. The survival depends on the treatment and the stage at diagnosis. In a study of 251 patients, the 5-year survival rate was 97% for those who had no positive lymph nodes on histological examination. In contrast the 5-year survival was 52% in those who presented with positive lymph nodes [1]. Shalhub *et al*, reported on a case of metastatic Merkel cell cancer. In their case the patient showed a lymph node in the axilla and in the groin. At the same time a CT scan showed multiple enlarged portocaval and gastrohepatic lymph nodes as well as a small lesions in the vicinity of the colon, which were metastatic Merkel cell carcinoma on histological examination [13].

Aggressive therapy of the primary tumor is crucial for improved outcomes. Surgery and adjuvant radiotherapy provide good local control of the primary tumor [5]. A retrospective study of 35 patients suffering from Merkel cell carcinoma showed that wide excision, lymph node dissection and adjuvant radiotherapy decreased the loco-regional recurrence, and patients had a better survival [14]. In a review of 1024 patients, the recurrence rate was 10.5% in those who received radiotherapy in comparison to 52.6% without radiation following resection [5].

### Pancreatic Metastases from Merkel Cell Cancer

The majority of the neoplasms in the pancreas have their origin in the organ itself. In a study examining 973 surgical specimen, only 38 metastatic tumors to the pancreas were noted [15]. The origin of pancreatic metastases were lymphomas, carcinomas of stomach, kidney, lung and others, as well as a Merkel cell carcinoma [15]. A Medline search with the key words *Merkel cell cancer, Merkel cell carcinoma, metastatic disease, pancreatic metastases and surgical treatment *revealed two additional reports of pancreatic metastasis from a Merkel cell carcinoma: One patient had a 4 cm violaceous firm subcutaneous nodule of the left lower eyelid. The remaining findings on physical examination were unremarkable. A biopsy confirmed that as Merkel cell tumor. Later this patient presented with jaundice and a cystic lesion (approximately 5 cm in diameter) in the pancreas. Subsequent laparoscopy showed evidence of peritoneal carcinomatosis, which was histological confirmed as a metastasis of the primary Merkel carcinoma [7]. In another case report, a 64-year old man presented with a short history of jaundice. A CT scan of the abdomen showed a large 6 cm mass in the head of the pancreas. Histological examination revealed a metastasis of Merkel cell carcinoma approximately two years after resection of a Merkel cell carcinoma resected from his finger [8]. As regards the role of imaging in Merkel cell carcinoma, clinical information is still insufficient to fully appreciate the role of imaging in the management of this condition. A better imaging algorithm is expected with increased awareness and improved clinical understanding of this uncommon neoplasm. Currently, CT scan, along with magnetic resonance imaging, appears to be imaging modality of choice. Contrast enhanced computed tomography (CT) demonstrates high-attenuation adenopathy and soft-tissue nodules. Solid abdominal organs targeted by metastasis manifest as hypervascular lesions with ringlike enhancement [16,17].

Our case adds some more information to the existing pool of knowledge about this tumor. It is well documented that treatment principles of this rare tumor revolve around adequate surgical resection and the addition of radiotherapy [2,18]. In systemic disease, many combinations of chemotherapeutic agents have been used with poor results [19]; although in a recent study, Merkel cell carcinoma was chemosensitive but rarely chemocurable in patients with metastases or locally advanced tumors [20]. While this elderly lady was possibly subjected to palliative radiotherapy given the extent of her abdominal mass, her unrelenting symptoms and the fact that age over 60 years is a poor prognostic factor in Merkel cell carcinoma prompted us to consider a surgical resection in spite of her advanced age. However, the symptoms (severe hypoglycemia) were not related to the metastatic Merkel cell carcinoma, but to the concomitant insulinoma. While her symptoms completely resolved due to excision of the insulinoma, the R0 excision of the Merkel cell carcinoma could also be expected to provide survival benefit; however this remains to be seen on longer follow-up.

Interestingly, Fincher *et al *reported a Merkel cell carcinoma with a concomitant somatostatinoma. Our case is the second case of Merkel cell carcinoma with a concomitant endocrine tumor. As already speculated by Fincher *et al*, this "occurrence may represent a previously undescribed neuroendocrine tumor syndrome, and this possibility should be considered when either tumor is diagnosed" [21]. Modern molecular biological methods may help to elucidate whether common molecular alterations are present in these tumor types.

## Competing interests

The author(s) declare that they have no competing interests.

## Authors' contributions

**JB **collated the information, searched the literature and wrote the manuscript.

**FB **and **WH **contributed to the pathological aspects of the manuscript and helped in preparing the manuscript.

**JK **and **SVS **assisted in literature search and writing of the manuscript.

**MWB **and **HF **managed the patient and helped in preparing the manuscript and edited the final version with **JK**.

All authors read and approved the final version of the manuscript.
